# Factors Influencing Post-traumatic Stress Symptoms in Chinese Adolescents During the COVID-19 Pandemic

**DOI:** 10.3389/fpsyt.2022.892014

**Published:** 2022-05-31

**Authors:** Siyuan Ke, Li Sun, Jiawei Zhou, Yini Wang, Tianyi Bu, Haiyun Chu, Jiarun Yang, Wenbo Wang, Wantong Wang, Jiarui Li, Lu Zhao, Zhengxue Qiao, Xiaohui Qiu, Xiuxian Yang, Depin Cao, Yanjie Yang

**Affiliations:** ^1^Department of Medical Psychology, Harbin Medical University, Harbin, China; ^2^Department of Medical Education Management, School of Health Management of Harbin Medical University, Harbin, China; ^3^The Second Affiliated Hospital of Harbin Medical University, Harbin, China

**Keywords:** COVID-19, adolescents, PTSS, family factors, home quarantine

## Abstract

To investigate the prevalence of post-traumatic stress symptoms (PTSSs) and analyze the influencing factors of PTSS among adolescents in a large sample study during the COVID-19 pandemic, we did a cross-sectional study by collecting demographic data and mental health measurements from a large group of 175,318 adolescents in 32 Chinese provinces and autonomous regions, using the Impact of Event Scale-Revised (IES-R) that was used to measure the PTSS of the participants. The results showed that the prevalence of PTSS was 35.7% in Chinese adolescents during the COVID-19 pandemic. Binary logistic regression analysis showed that, for the personal risk factors, the older age, female gender, the personality domains of extroversion, the irregular sleep schedule, the lack of aerobic exercise, and the lack of peer support were associated with the higher levels of PTSS. The family subjective and objective factors were associated with higher levels of PTSS. Our findings suggested that family factors are the most important factors that affect Chinese adolescents' PTSS due to the longtime home quarantine.

## Introduction

On 30 January 2020, the World Health Organization declared the 2019 novel coronavirus disease (COVID-19) as a public health emergency of international concern (1). COVID-19 has swept across 210 countries and territories with over 92 million cases and 479,133 deaths reported by 25 June 2020 (2). Most countries have implemented social isolation measures, such as school closures and some public places, to curb the spread of infection and minimize the impacts of the virus (3). According to United Nations Educational, Scientific, and Cultural Organization (UNESCO), by 8 April 2020, schools in 188 countries around the world have closed due to COVID-19 (4). To halt the spreading of COVID-19 in schools, China's ministry of education has asked for a postponement of the spring semester in 2020. This move has led to more than 220 million children and adolescents confined to their own homes in China (5).

Physical damages caused by public health emergencies have been pointed out that could be recovered in a short time, but the psychological impacts will last for a long time (6). Teenagers are in adolescence, with immature psychological mechanisms, which makes them more susceptible to changes in the external environment and prone to psychological problems (7). Due to the incomplete cognitive development of adolescents, they have limited knowledge about the epidemic situation, which may lead to negative emotions, such as fear and anxiety. Adolescence is usually associated with increased risk-taking behaviors, increased needs for social connection and peer acceptance, and increased sensitivity to peer influence (8). During this period, young people grow in independence and begin to prioritize connections with peers over parents (9). Due to home quarantine, the need for adolescents to get along with their peers is difficult to be met and they have to spend more time with their parents. So, this means that the family environment plays an important role in the psychological development of adolescents and has a great impact on them during the outbreak (10). A good parent–child relationship and family atmosphere can positively promote the personality development and mental health of adolescents. In the face of COVID-19, support and care from parents can help adolescents to overcome negative emotions and protect their psychological resilience. However, many parents in China have “Chinese parental authority” in the way they communicate. Such excessive parental control or overprotection can backfire on a child who is in adolescent rebellion. Parent–adolescent conflict is regarded as the “major influence” in adolescent unacceptable behavior (11). In addition, as examinations are still covered during the pandemic, adolescents are likely to have concerns about their studies and future development. For these adolescents who may need mental health support, such suspensions at home mean that they do not have access to the resources normally available through schools and some social supports. Furthermore, changes in the study method, such as online learning and independent reading, could bring more challenges for young people (12). In conclusion, the interaction between lifestyle changes brought about by home confinement and the pandemic itself could further exacerbate the detrimental effects on adolescents' mental health and may even lead to a vicious circle (13).

Adolescents face stresses, such as prolonged duration, fears of infection, lack of in-person contact with peers, and reduced personal space at home, may appear even more problematic and enduring symptoms (14). These symptoms manifest as the repeated experience of invasion, avoidance, and increased alertness, which are closely affiliated with post-traumatic stress disorder (PTSD) symptoms (15). When people face major stress events, such as interpersonal conflicts, deaths of family members, traffic accidents, natural disasters, and disease epidemics, there will be a series of post-traumatic stress symptoms (PTSSs) (16). PTSSs are the result of multiple factors, including stress, genetics, personality, coping style and social support, past trauma experience, past mental illness, and so on (17). Usually, this reaction may be temporary and controllable. Once they cannot solve it, serious psychological imbalances may occur, and some people will develop PTSD and related symptoms (18). However, if the individual is found in a timely manner and can receive appropriate post-traumatic psychological support, it is possible to avoid or reduce PTSD. There is an increasing number of evidence showing that the Impact of Event Scale—Revised (IES-R) is valid and reliable as “a low-cost measure to detect PTSD” (19). The IES-R is a screening tool rather than a formal diagnostic test, but it correlates well with the diagnosis of PTSD (20). The IES-R is a self-report measure for capturing the level of symptomatic responses to specific traumatic stress in the past week (21). The IES-R can timely assess the individual's PTSS level after the traumatic event, so as to effectively screen the PTSD (22).

This study represents the survey based on a large sample of adolescents in the mainland of China and aims to (1) describe the prevalence of PTSS among adolescents during the pandemic and explore whether the severity of the pandemic will affect the prevalence of PTSS and (2) investigate and analyze the influencing factors of adolescents' PTSS during the COVID-19 pandemic, so as to provide a theoretical basis for reducing the prevalence of PTSD in adolescents worldwide.

## Methods

### Study Design and Participants

Our online cross-sectional survey was conducted in the mainland of China from February 23 to March 8, 2020. The Chinese adolescents aged 16–26 years were invited to participate in the online survey through the Survey-Star platform, and the electronic questionnaire was distributed through the WeChat group and encouraged to pass it on to others. We carefully check the quality of each questionnaire to ensure that the returned questionnaire is filled in effectively. This web-based questionnaire was completely voluntary and non-commercial. A total of 175,318 adolescents covered all 32 provinces and autonomous regions have completed the questionnaires in this study during the COVID-19 pandemic. This study was approved by the Ethics Committee of Harbin Medical University (HMUIRB20200002). The attributes, benefits, uses, and disadvantage effects of the study were explained to all the participants. Each participant provided online informed consent to participate in the study.

### Procedures

The adolescents completed the following scale. The Impact of Event Scale-Revised (IES-R) was used to measure the post-traumatic stress symptoms of the participants (23). The IES-R is a self-report questionnaire and consisted of 22 items, each item scored from 0 (no problems) to 4 (frequent problems). The total score is obtained by summing across all items, which ranges from 0 to 88. The cutoff score of IES-R is 20, and a score of ≥20 on the IES-R is used to estimate the prevalence of PTSS, with higher IES-R scores indicating more symptoms (24). The scale had 3 subscales: avoidance (8 questions), intrusion (8 questions), and hyperarousal (6 questions). IES-R has previously been shown to have good reliability and has been widely used among the Chinese population. In this study, the Cronbach's α value for all 22 items was 0.964.

Sociodemographic data included the participant's gender (male/female), age (16–20/21–26), residential areas (rural/urban), situation of COVID-19 epidemic in residence whether cumulative confirmed cases >500 (slight/severe), symptoms related to COVID-19 during past 14 days included fever, cough, and fatigue (no/yes), relatives or friends having symptoms related to COVID-19 (no/yes), personality type (introvert/general/extrovert), sleep time (<5 h/5–7 h/8–10 h), peer support (no/yes), aerobic exercise >30 min per day during the epidemic (no/yes), having a separate room at home (no/yes), family economic status (poor/general/good), relationships with family (good/general/poor), parental education (senior high school and below/junior college and above), quarrel with parents >3 times during home quarantine (no/yes), overprotection from parents (no/yes), and activities with parents during home quarantine (no/yes).

### Statistical Analysis

The Statistical Package for Social Science 22.0 (SPSS 22.0) program was used for all statistical analysis. Continuous data are shown as mean (standard deviation). To examine the differences in demographic, psychological, and COVID-19 epidemic-related variables, we conducted the chi-square tests, the *t*-tests, and the F-tests. Binary logistic regression analysis was used to identify the factors associated with PTSS, independent variables include age, gender, residential areas, situation of COVID-19 epidemic in residence, symptoms related to COVID-19 during the past 14 days, relatives or friends having symptoms related to COVID-19, personality type, exercise during the epidemic, peer support, family economic status, relationship with family, sleep time, separate room, quarrels between parents during home quarantine, parental education, overprotection from parents, and activities with parents. *p*-Values < 0.05 were regarded as significant for all tests (two-sided).

## Results

A total of 175,318 valid questionnaires had been collected, including 80,595 (46.0%) men and 94 723 (54.0%) women. The average age of the study population was 20.52 ± 1.69 years (mean ± SD), ranged from 16 to 26 years. Table 1 shows the means and standard deviations of the IES-R subscale scores, and the mean score for IES-R was 32.80 ± 11.69. The results showed that 35.7% of the students had a total IES-R score ≥ 20, that is to say, the prevalence of PTSS among Chinese adolescents was found to be 35.7%. Furthermore, Table 1 presents the scores of each dimension among adolescents who experience PTSS. The dimensions with the highest means were avoidance, intrusion, and hyperarousal.

**Table 1:** PTSS, scores, and dimensions in the adolescents who scored ≥ 20 (*N* = 62,668).

**Variables**	**Range**	**Mean (SD)**	**Mean item score**
Avoidance	0–32	12.53 (4.80)	1.56
Intrusion	0–32	11.61 (4.60)	1.45
Hyperarousal	0–24	8.65 (3.81)	1.44
IES total score	20–88	32.80 (11.69)	1.49

Additionally, Table 2 displays the basic demographic data, IES-R scores, and the prevalence of PTSS (IES-R score ≥ 20) for each demographic group. The results showed that there were significant differences in PTSS related to gender, age, residential areas, situation of COVID-19 epidemic in residence, symptoms related to COVID-19 during the past 14 days, relatives or friends having symptoms related to COVID-19, personality type, sleep time, peer support, exercises during the epidemic, having a separate room at home, family economic status, relationships with family, parental education, quarrels with parents, overprotection from parents, and activities with parents.

**Table 2:** Socio-demographic factors for the sample of Chinese adolescents.

**Variables**	**Group**	***N*** **(%)**	**IES-R score**	***F***/***t***	**IES-R score ≥20 (%)**
Gender	Male	80,595 (46.0)	14.33 (16.21)	−23.35^**^	34.0
	Female	94,723 (54.0)	16.07 (14.88)		37.3
Age	16–20	93,454 (53.3)	15.26 (15.48)	−0.39^**^	35.3
	21–26	81,864 (46.7)	15.29 (15.58)		36.2
Residential areas	City	70,254 (40.1)	15.61 (15.28)	7.59^**^	36.9
	Rural	105,064 (59.9)^*^	15.05 (15.68)		35.0
Situation of COVID-19 epidemic in residence	Severe	50,736 (28.9)	16.11 (16.01)	14.45^**^	37.6
	Slight	124,582 (71.1)	14.93 (15.31)		35.0
Having symptoms related to COVID-19	Yes	1,073 (0.6)	26.11 (20.66)	22.97^**^	60.2
	No	174,245 (99.4)^*^	15.20 (15.47)		35.6
Relative or friends having symptoms related to COVID-19	Yes	1,573 (0.9)	22.51 (18.93)	18.57^**^	53.5
	No	173,745 (99.1)^*^	15.21 (15.48)		35.6
Personality type	Extrovert	35,587 (20.3)	16.89 (16.21)	261.42^**^	39.5
	Ambivert	94,572 (53.9)	15.04 (15.22)		35.4
	Introvert	45,159 (25.8)	14.49 (15.52)		33.4
Exercise during the epidemic	Yes	127,735 (72.9)	15.30 (15.16)	1.105^**^	34.9
	No	47,583 (27.1)	15.20 (16.46)		36.1
Sleep time	<5 h	35,015 (20.0)	16.61 (17.00)	176.4^**^	39.0
	5–7 h	104,757 (59.8)	15.07 (14.78)		35.6
	8–10 h	35,546 (20.2)	14.56 (16.07)		33.0
Peer support	Yes	137,855 (78.6)	14.81 (14.92)	−23.72^**^	34.6
	No	37,463 (21.4)	16.96 (17.48)		40.0
Separate room	Yes	17,572 (10.0)	22.61 (18.43)	66.82^**^	52.5
	No	157,746 (90.0)	14.45 (14.95)		33.9
Family economic status	Good	29,802 (17.0)	15.42 (15.85)	5.89^**^	35.5
	Moderate	116,516 (66.5)	15.18 (15.32)		35.7
	Poor	29,000 (16.5)	15.48 (15.99)		36.2
Relationships with family	Good	167,431 (95.5)	14.91 (15.24)	1,191.34^**^	34.9
	Moderate	7,225 (4.1)	22.09 (18.27)		52.2
	Poor	662 (0.4)	33.04 (23.78)		67.2
Parental education	Senior high school and below	85,031 (48.5)	16.10 (15.82)	21.68^**^	38.1
	Junior college and above	90,287 (51.5)	14.49 (15.20)		33.6
Quarrels between parents during home quarantine	Yes	72,527 (41.4)	16.46 (16.04)	26.98^**^	39.0
	No	102,791 (58.6)	14.43 (15.09)		33.4
Overprotection from parents	Yes	24,054 (13.7)	21.02 (17.98)	62.76^**^	50.0
	No	151,264 (86.3)	14.36 (14.90)		33.5
Activities with parents	Yes	99,963 (57.0)	14.98 (15.03)	−9.14^**^	36.8
	No	75,355 (43.0)	15.66 (16.15)		34.9

* *P < 0.05*.

** *P < 0.01*.

**Figure 1:**
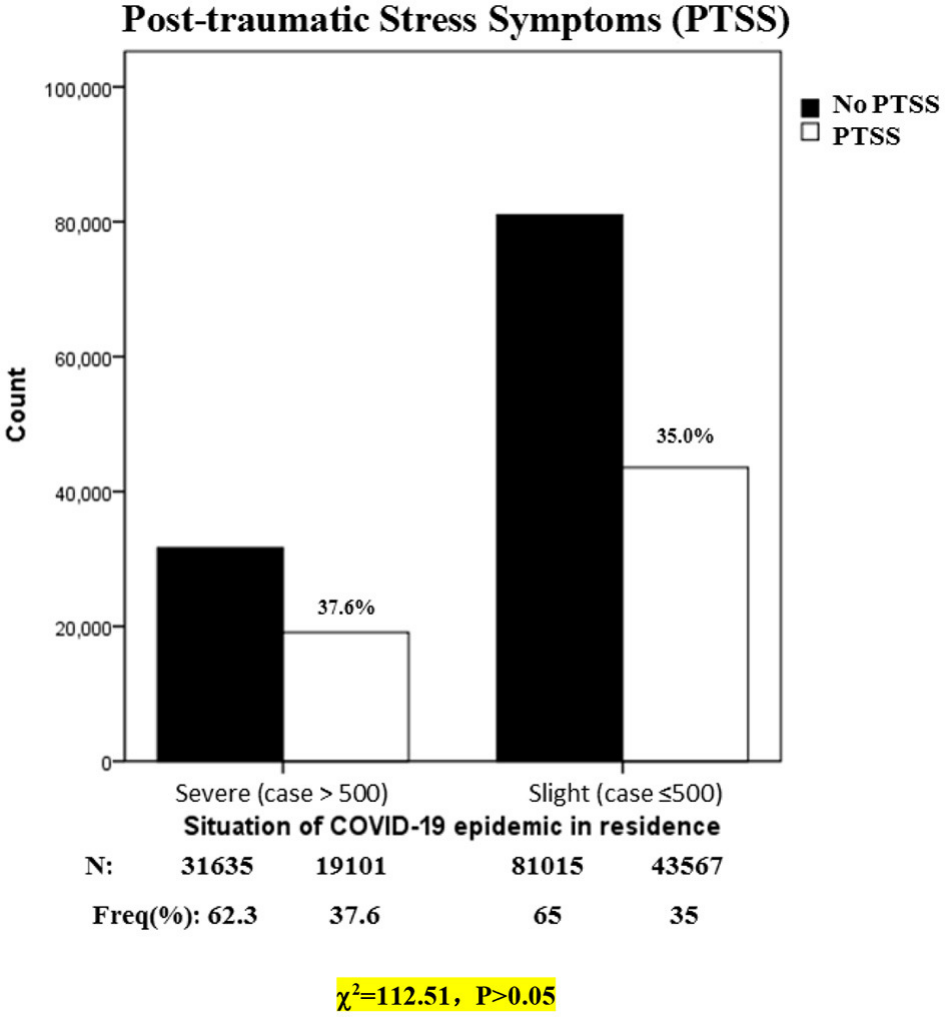
Prevalence of PTSS during COVID-19 pandemic in China, stratified by situation of COVID-19 epidemic in residence (*N* = 175,318). N, number; Freq, frequency.

Figure 1 shows that the prevalence of PTSS was 35.0% in slight epidemic areas and 37.6% in severe areas. The prevalence of PTSS between slight and severe epidemic areas was small and almost identical. Therefore, the prevalence stratified by the situation of COVID-19 epidemic in residence could be considered as no differences.

Table 3 presents the binary logistic regression analysis results for the PTSS-dependent variables. Although the analysis indicated that all the independent variables were associated with PTSS, some independent variables' odds ratio values were approached to 1.00, including age [odds ratio (OR) = 1.011, 95% confidence interval (CI) = 1.005–1.017], residential areas (OR = 0.931, 95% CI = 0.912–0.915), situation of COVID-19 epidemic in residence (OR=0.921, 95% CI =0.900–0.941), exercise during the epidemic (OR = 1.060, 95% CI = 1.035–1.084), parental education (OR = 1.161, CI = 1.138–1.185), and family economic status. Those variables that OR value approached to 1.00 were weakly associated with PTSS; meanwhile, the other independent variables had a stronger impact on PTSS. The results identified that having symptoms related to COVID-19 during the past 14 days (OR = 2.302, 95% CI = 2.028–2.612), relatives or friends having symptoms related to COVID-19 (OR = 1.922, 95% CI = 1.735–2.128), overprotection from parents (OR = 1.689, 95% CI = 1.640–1.738), quarrels with parents during home quarantine (OR = 1.254, 95% CI = 1.229–1.280), gender (OR = 1.203, 95% CI = 1.179–1.228), good relationship with family (OR = 0.487, CI = 0.411–0.577), separate room (OR = 0.518, 95% CI = 0.501–0.535), peer support (OR = 0.798, 95% CI = 0.779–0.818), introvert personality type (OR = 0.846, 95% CI = 0.821–0.871), sleep 8–10 h (OR = 0.860, 95%CI = 0.833–0.888), and activities with parents (OR = 0.897, 95% CI = 0.879–0.916) were the significant predictors for PTSS in adolescents. Having symptoms related to COVID-19 during the past 14 days played the most important risk role in PTSS, and a good relationship with family played the most important protective role in PTSS.

**Table 3:** Binary logistic regression: the risk and protect factors of PTSS.

**Variables**	***B***	**S.E**.	**Exp (*B*)**	**95% C.I.for EXP (** ***B*** **)**		**Tol**	**VIF**
				**Lower**	**Upper**		
Age	0.011	0.003	1.011^**^	1.005	1.017	0.995	1.005
Gender	0.185	0.010	1.203^**^	1.179	1.228	0.967	1.034
Residential areas	−0.071	0.011	0.931^**^	0.912	0.951	0.977	1.024
Situation of COVID-19 epidemic in residence	−0.083	0.011	0.921^**^	0.900	0.941	0.994	1.006
Symptoms related to COVID-19 during past 14 days	0.834	0.065	2.302^**^	2.028	2.612	0.992	1.008
Relatives or friends having symptoms related to COVID-19	0.653	0.052	1.922^**^	1.735	2.128	0.991	1.013
Personality type						0.968	1.033
Ambivert	−0.102	0.013	0.903^**^	0.880	0.927		
Introvert	−0.168	0.015	0.846^**^	0.821	0.871		
Exercise during the epidemic	0.058	0.012	1.060^**^	1.035	1.084	0.990	1.010
Peer support	−0.226	0.013	0.798^**^	0.779	0.818	0.955	1.047
Family economic status						0.966	1.036
General	0.080	0.014	1.083^**^	1.054	1.113		
Poor	0.102	0.018	1.107^**^	1.069	1.146		
Relationship with family						0.931	1.074
General	−0.363	0.089	0.696^**^	0.584	0.829		
Poor	−0.719	0.087	0.487^**^	0.411	0.577		
Sleep time						0.950	1.053
5–7 h	−0.040	0.013	0.961^**^	0.936	0.986		
8–10 h	−0.151	0.016	0.860^**^	0.833	0.888		
Separate room	−0.658	0.017	0.518^**^	0.501	0.535	0.943	1.037
Quarrels between parents during home quarantine	0.226	0.010	1.254^**^	1.229	1.280	0.954	1.048
Parental education	0.150	0.010	1.161^**^	1.138	1.185	0.972	1.028
Overprotection from parents	0.524	0.015	1.689^**^	1.640	1.738	0.900	1.111
Activities with parents	−0.108	0.011	0.897^**^	0.879	0.916	0.919	1.088
Constant	1.123	0.115	3.074				

** *P < 0.01*.

## Discussion

This survey was conducted during the rapid rising period of the COVID-19 pandemic, and the adolescent population covered 32 provinces and autonomous in the mainland of China. The results illustrated that the prevalence of PTSS was 35.7% in Chinese adolescents during the COVID-19 pandemic, and avoidance symptoms were of the highest score among all the three symptoms in adolescents with PTSS. As the previous study reported, about 14.5–21.1% of children and adolescents were diagnosed with PTSS during the month after being exposed to traumatic events, such as earthquakes and snowstorms (25). These unprecedented challenges have been reported to cause more than half of the participants (52.1%) feeling horrified and apprehensive (26), which may result in increased PTSS prevalence and high avoidance symptoms in our study. Therefore, to improve the adolescent's mental health status over the long term, there is a need to study how the pandemic itself prolonged and strict social distancing measures affect the PTSS of adolescents.

In our analysis, it is surprised to find that the family environment was the most important factor of the PTSS of Chinese adolescents during the COVID-19 pandemic. Due to the home quarantine, adolescents will spend more time with families which will make the role of family factors become particularly important. Family environmental factors could be divided into objective factors and subjective factors. Family objective environmental factors basically are to point to family material condition environment (27), which include parental education and occupation, family structure, family economic status, etc. The family subjective factors include the relationship with families, parental relationship, parenting style, communication with parents, etc. Subjective factors are mainly social environment within the family, which has been considered as an important factor regarding the PTSS of adolescents (28).

Among the family objective environmental factors, the strongest indicator of the PTSS was whether the adolescent has a separate room at home. We found that adolescents without separate rooms are associated with higher levels of PTSS. Anderson et al. illustrated that adolescents have more needs for privacy than younger children (29). During adolescence, the desire to seek personal independence and privacy protection increases (30). For adolescents without a separate room, it may be easier for parents or family members to take direct boundary invasion that includes interrogating, giving unsolicited advice, violating private space, and so forth. Due to COVID-19, adolescents spent more time at home with a lack of private time and space. In response, adolescents had to take defensive actions, such as evading or confronting, meanwhile, psychological pressure increases accordingly (31). This puts them at a much higher risk of developing PTSD. Although the independent room provides a certain “spatial distance” for adolescents and their parents, the generation of “spatial distance” also largely avoids the trivial collision and friction in daily life. It greatly improves the private family life and maintains the emotional exchange and support between the members of the extended family.

Compared with these objective factors, the subjective factors from the family in the study were found to be more important and controllable. The family subjective environmental factors were a major factor in the prevalence of PTSS among Chinese adolescents. Poor relationship with family, overprotection from parents, parents arguing more, and no joint activities with parents were associated with higher levels of PTSS. It was found that adolescents with poor family relationships are more prone to PTSS. The evidence summarized in Klein's paper indicated that the nature and quality of relationships with family members matter profoundly for all adolescents, and perhaps uniquely for adolescents who experience traumatic events (32). Keeshin and Strawn study also suggested that good parent–child relationships are beneficial for reducing PTSD in adolescents (33). These strongly support our conclusions. It is no doubt that adolescents who have a good relationship with their families tend to have more support from their families. Family support is crucial for adolescents who stay in isolation. Anna's study proved that family support, such as love, care, and attention, is important and beneficial for all adolescent victims of disasters (33). However, low cohesion families usually were reluctant to offer help and support. Individuals from these families will increase the likelihood to suffer PTSD because they are more likely feeling completely alone when they face stress. Due to China's unique one-child policy, the current structure of most adolescents' families is composed of parents and only child. This allows parents to put more attention and expectation on the only child, which leads to excessive protection from parents. Moreover, parental overprotection is particularly important for the prevalence of PTSS in adolescents based on regression results. It has been reported that overprotection from parents in combination with higher levels of exposure to trauma was associated with the highest levels of PTSD symptoms among adolescent victims of disaster (34). Another study has also pointed out that defects in parenting, such as overprotection by parents, a lack of warmth, and effective response, can easily lead to adverse cognitive patterns in children (35). These conclusions are consistent with our views. Such excessive protection may make children believe that they cannot control the occurrence or outcome of everything, aggravate their psychological burden of the epidemic situation, and maintain it for a long time, thus resulting in PTSS. As Hobfoll's (1988) COR theory suggested that declines in the perceptions of increases in interpersonal negativity in the family (e.g., greater frequency of family quarrels) are losses in the family resources that determine adolescents' experience of stress and its deleterious consequences (36). In our study, parental quarrels increased during the period of staying at home, and the increased conflict would increase the degree of PTSS of adolescents. A previous study has confirmed that adolescents with high parental quarrels showed higher levels of PTSD symptoms 2 years after the traumatic event (34). This is consistent with our findings. As a result of the home quarantine, adolescents' parents spent more time at home than before. Strains on household expenses, fears about the epidemic, household chores, and inconsistencies in children's education all come to the fore at home and lead to arguments between couples. Quarrels between parents cannot only directly affect adolescents' immediate emotional problems, such as depression, anxiety, and hostility (37), but also indirectly cause chronic stress responses in adolescents through their perception of hostility and quarrels between parents. In addition, joint activities with parents during the epidemic are the protective factors for adolescents' PTSS. Family activities, such as eating with family, watching TV, and doing household chores, are very helpful to enhance the overall family atmosphere (38). The overall family environment and atmosphere can greatly help adolescents to resist external traumatic stress events. Thus, adolescents and their families are encouraged to carry out beneficial family activities during segregation at home. It is conducive to enhance the emotional communication between parents and children and strengthen the psychological resilience of adolescents.

Based on the above analysis, some suggestions have been provided. First, during the epidemic, parents should give more family support to adolescents and adopt proper parenting methods. Adolescents need to be given enough trust and space to develop. In addition, parents were suggested to stick to the principles when it comes to educating the children and not control everything in the name of “love.” While creating space for adolescents, they try to help them overcome negative emotions and maintain emotional stability. Moreover, when there are quarrels and differences between couples, to communicate in a timely manner, they face together and create a comfortable and peaceful family atmosphere for children. Furthermore, adolescents were encouraged to actively maintain good communication with parents, consciously improve their mental resilience, and maintain a good family relationship.

### Study Limitations

It is important to recognize the limitations of this survey. First of all, this survey is a cross-sectional study, and future prospective studies are needed to determine correlation and causation. Second, although the survey conducted online is suitable for rapid assessment, there may be some bias in the sample that reduces the ability of the results to be generalized. Finally, PTSSs were assessed through self-report questionnaires only, which may not be as accurate as face-to-face interviews to allow a diagnosis of clinically relevant disorders.

## Conclusion

To the best of our knowledge, our study established the largest Chinese adolescent cohort to investigate the impacts of the COVID-19 pandemic on PTSS. The findings showed that there was a high prevalence of PTSS in adolescents during the pandemic. One interesting and important phenomenon was that the extents of PTSS in the slight and severe areas were almost the same. The influencing factor analysis indicated that it was not only the epidemic itself, but also the personal characteristics, health behavior, and peer support which were associated with PTSS. Among these factors, the family environment factors took the strongest influence on PTSS prevalence, which included the subjective environmental factors, such as the poor family relationship and overprotection from parents, and the objective environmental factors, such as lack of private space. The results highlight that the parents should enhance the interaction with adolescents and give adolescents more respect to comfort them in prolonged isolation, meeting their needs for private spaces to minimize the PTSS of the COVID-19 pandemic on adolescents. Above all, our study can help to have a better understanding of how the epidemic affects adolescent's mental health and offered valuable strategies to ameliorate the PTSS caused by viral pandemics in adolescents.

## Data Availability Statement

The raw data supporting the conclusions of this article will be made available by the authors, without undue reservation.

## Ethics Statement

The studies involving human participants were reviewed and approved by the Ethics Committee of Harbin Medical University (HMUIRB20200002). The patients/participants provided their written informed consent to participate in this study.

## Author Contributions

YY, DC, and SK conceived, designed the study, and interpreted the data. XY, JZ, and LS supervised the analysis, interpreted the data, and wrote the preliminary manuscript. TB, HC, and YW supervised the data collection by JY, WaW, JL, and WeW. LZ, ZQ, and XQ compiled the data. All authors contributed to the writing and review of the manuscript and approved the final version.

## Funding

This research was supported by the National Natural Science Foundation of China (81773536) by YY.

## Conflict of Interest

The authors declare that the research was conducted in the absence of any commercial or financial relationships that could be construed as a potential conflict of interest.

## Publisher's Note

All claims expressed in this article are solely those of the authors and do not necessarily represent those of their affiliated organizations, or those of the publisher, the editors and the reviewers. Any product that may be evaluated in this article, or claim that may be made by its manufacturer, is not guaranteed or endorsed by the publisher.
